# Longitudinal Relationships Between Favorite Activities and Homebound Status in Older Adults

**DOI:** 10.1093/geroni/igaa057.1603

**Published:** 2020-12-16

**Authors:** Xiaocao Sun, Minhui Liu, Christina Miyawaki, Yuxiao Li, Tianxue Hou, Siyuan Tang, Sarah Szanton

**Affiliations:** 1 Central South University, Changsha City, Hunan, China; 2 Central South University, Baltimore, Maryland, United States; 3 University of Houston, Houston, Texas, United States; 4 Central South University, Changsha, Hunan, China; 5 Johns Hopkins University, Baltimore, Maryland, United States

## Abstract

Favorite activities are usually meaningful to older adults and may influence their homebound status and vice versa. Using Round 1 (R1, in 2011) and Round 5 (R5, in 2015) data from the National Health and Aging Trends Study, we examined the patterns of favorite activity by homebound status and investigated their relationship among community-dwelling older adults (N=3,332). Homebound status (non-homebound, semi-homebound, and homebound) was determined by the frequency, difficulty, and needing help of outdoor mobility. Favorite activities were named by participants verbatim and then were classified into two categories (active and non-active) based on the estimated energy needed to perform the activity. Logistic regression models were used to determine whether homebound status at R1 predicted the types of favorite activity in R5, and ordinal logistic regression models for predictions from the types of favorite activity at R1 to homebound status at R5, adjusting for demographics and health conditions. The sample was, on average, 76±7.23 years old, non-Hispanic White (72.9%), and female (59.7%). Regardless of the homebound status, reading and walking are the top two favorite activities. Homebound older adults enjoyed “non-active” activities (e.g., watching TV), while non-homebound counterparts preferred “active” outdoor maintenance. Being homebound at R1 predicted non-active favorite activity in R5 (OR=.257, p<0.001), and R1 non-active favorite activity also predicted homebound status in R5 (OR=1.219, p =0.039). These findings provide new information on the activity preferences of older adults with different homebound status and how their preferences may influence their future homebound status.

